# Supplementary material for: Rapid turnover of life-cycle-related genes in the brown algae
Source: Genome Biol. 2019 Feb 14;20:35. doi: 10.1186/s13059-019-1630-6 (PMC6374913; doi:10.1186/s13059-019-1630-6)
Supplement: Supplementary file 3 — Protein alignments, corresponding Gblocks html file and CDS sequences for the lineage conserved gametophyte-biased genes. (ZIP 2109 kb) [file 13059_2019_1630_MOESM3_ESM.zip › Con_GA_alignments/Ec-21_000880.aa_ali.fasta-gb.htm]

Ec-21\_000880.aa.fasta.trans.aa\_ali.fasta


## Gblocks 0.91b Results

Processed file: **Ec-21\_000880.aa.fasta.trans.aa\_ali.fasta**  
Number of sequences: **2**  
Alignment assumed to be: **Protein**  
New number of positions: **602** (selected positions are underlined in blue)

```
                         10        20        30        40        50        60
                 =========+=========+=========+=========+=========+=========+
Ec-21_000880     MAVRRDGMTIANVNSFLEMSGADREAGEIVNAAGIKISDPVQEPGYGRTVDVVKLREDLL
MSTRG.40198.1    ------------------------------------------------------------
                                                                             


                         70        80        90       100       110       120
                 =========+=========+=========+=========+=========+=========+
Ec-21_000880     LKLQNERSEEVGGLRAKQKQIMSDKKGREAELLELQKAEESHRELLLKDRDERNKIAQDL
MSTRG.40198.1    ------------------------------------------------------------
                                                                             


                        130       140       150       160       170       180
                 =========+=========+=========+=========+=========+=========+
Ec-21_000880     HCLRRSHLVDMEEETRRELASLQRERELLQEKEKNNEAAIDEVRRKIMEQDKVYHHAMDA
MSTRG.40198.1    ------------------------------------------------------------
                                                                             


                        190       200       210       220       230       240
                 =========+=========+=========+=========+=========+=========+
Ec-21_000880     ARSKIKTGKNGVDQDGRPMADTARSQRQAIQSEQVAIAEVYGKRAGTLQEEQTRLQHEIQ
MSTRG.40198.1    ------------------------------------------------------------
                                                                             


                        250       260       270       280       290       300
                 =========+=========+=========+=========+=========+=========+
Ec-21_000880     SVDERIQKARKNGLAPAKGADDALAALMTEAGMEEQATRLRRLKGDFEKQHAEDVSGAFI
MSTRG.40198.1    ------------------------------------------------------------
                                                                             


                        310       320       330       340       350       360
                 =========+=========+=========+=========+=========+=========+
Ec-21_000880     KDYEGVEAKTRKALKNSAKLLEGRTGEYAEATADAFLDDWLKTDADRAREAAAAAAAAAA
MSTRG.40198.1    ------------------------------------------------------------
                                                                             


                        370       380       390       400       410       420
                 =========+=========+=========+=========+=========+=========+
Ec-21_000880     GGRLLSPHDNIPAIDRTIGAASTGADATGQGGVGRRLSATRNGTTAHEGHKKKRRSSRDR
MSTRG.40198.1    ------------------------------------------------------------
                                                                             


                        430       440       450       460       470       480
                 =========+=========+=========+=========+=========+=========+
Ec-21_000880     GHGNGHYTRRRSSGGRKIHRKGRTSRDSAGTSSAGSSDSEGGRSNDHVAGHGVSHGRHGG
MSTRG.40198.1    ------------------------------------------------------------
                                                                             


                        490       500       510       520       530       540
                 =========+=========+=========+=========+=========+=========+
Ec-21_000880     VVGGQGGNQQRRRNAAVGPISPPNGVAPAAPADSQPSAAEHHHHRNRAGEHRAPNERLAG
MSTRG.40198.1    ------------------------------------------------------------
                                                                             


                        550       560       570       580       590       600
                 =========+=========+=========+=========+=========+=========+
Ec-21_000880     EHRSDKSGEGGSDGGDARPAAQQPESISFGQAGREDEEQESLRRAKDDSAQLRATVERLE
MSTRG.40198.1    ------------------------------------------------------------
                                                                             


                        610       620       630       640       650       660
                 =========+=========+=========+=========+=========+=========+
Ec-21_000880     RELSSVKAGTRMGAGGVVGAQGEGFGLAGGSGGSRWGPPTDTTAGAPWMAGGMAAPGLQQ
MSTRG.40198.1    ------------------------------------------------------------
                                                                             


                        670       680       690       700       710       720
                 =========+=========+=========+=========+=========+=========+
Ec-21_000880     YLASLDAENARMMAEIQGLSSGGAGGGGQQFGLTGLGGDANVGLSTMFPGPAGATAGKGM
MSTRG.40198.1    ------------------------------------------------------------
                                                                             


                        730       740       750       760       770       780
                 =========+=========+=========+=========+=========+=========+
Ec-21_000880     AAAGYLGPGLHGNGRGPQVDGSGEQGEGGGGSAITAARKRSRVNGEKERVGPEWDEELPE
MSTRG.40198.1    ------------------------------------------------------------
                                                                             


                        790       800       810       820       830       840
                 =========+=========+=========+=========+=========+=========+
Ec-21_000880     AGGEPRSPRNKEYRRKMIEMDAEHKEAVKRLEFEMQRLELEQQLEDMRERMAREKKTRQQ
MSTRG.40198.1    ------------------------------------------------------------
                                                                             


                        850       860       870       880       890       900
                 =========+=========+=========+=========+=========+=========+
Ec-21_000880     DVAHEQWVTEQKRNLQAIKIQKTLAKERQLIEVQQTLTDARGGGVGNGALGGGRLAGGGF
MSTRG.40198.1    ------------------------------------------------------------
                                                                             


                        910       920       930       940       950       960
                 =========+=========+=========+=========+=========+=========+
Ec-21_000880     GEVAAALHAAGGWRPGVGGIVQRPYNKDQGFCVFWDYLTGLPKRSGSKVIGKVRLAFGLY
MSTRG.40198.1    ------------------GIVQRPYNKDKGFCVFWDYLTGLPKRSGSKIIGKVRLAFGLY
                                   ##########################################


                        970       980       990      1000      1010      1020
                 =========+=========+=========+=========+=========+=========+
Ec-21_000880     IGKRLHGKMLSTPWTDIAPRAVKNGNTGKAETFSKLETRSTFPGIPVHLQTKLVVELQFK
MSTRG.40198.1    TGKRLHGKMLSTPWTDIAPRPVKNETTGKPETFCKLETRSTFPGIPLHVQTKLVVELQFK
                 ############################################################


                       1030      1040      1050      1060      1070      1080
                 =========+=========+=========+=========+=========+=========+
Ec-21_000880     DEAGVQRKGGKGNLGWGCIYVFKPEKGSPHRLTLRAGLTRLTLFPGSVDLRIANVNDDKS
MSTRG.40198.1    DEAGVQAKGGKGNLGWGCFYVYKPEKGVPHRLTVRAGLTRLTLFPGSVDLRIANVNDDKS
                 ############################################################


                       1090      1100      1110      1120      1130      1140
                 =========+=========+=========+=========+=========+=========+
Ec-21_000880     AVSRSTPTAAFIRLVHGMDMNPAAAFVVDPDTQHLYAAYGRGNIPQMERQKLMAGTFLRA
MSTRG.40198.1    AVSRSTPTAAFIRLVHGEDMNPAGAFVVDPETQHLYTAYGRGNISQMERQKLMAGTFLRA
                 ############################################################


                       1150      1160      1170      1180      1190      1200
                 =========+=========+=========+=========+=========+=========+
Ec-21_000880     ILALGKFKRALVTHKRTRASGGDAAKDHPPLRRRSSSPLRRGSTRRTSFKLPRTGTFSSE
MSTRG.40198.1    ILIIGKLKRSLTSRKRVRASGVDAPKHAS--------PLQKSASRRGSFKARVTHAFTSP
                 ##########################           #######################


                       1210      1220      1230      1240      1250      1260
                 =========+=========+=========+=========+=========+=========+
Ec-21_000880     TRS--EPQAADGTDG-TDAEDGEGAKQPKADKQQASKPDEKRSESPKSEERDSVEEEDSE
MSTRG.40198.1    TANGLDLQADEAAAGITDTTDDARAKPVKASKHPPPEP-EKSTESSESDSGDSDEARDSE
                 #               ###################### #####################


                       1270      1280      1290      1300      1310      1320
                 =========+=========+=========+=========+=========+=========+
Ec-21_000880     -EDSGSAKGGGRE-TKERRRSSLSSIHSQLAGQKTMARRASLSKRRQSTVSRQPFLKGDG
MSTRG.40198.1    SDDEGSSEEDKRSGPMERRRSSLSSLHSQLAGQKTMAKRASLSKRRRSTISRQQFLKGDG
                   ##########    ############################################


                       1330      1340      1350      1360      1370      1380
                 =========+=========+=========+=========+=========+=========+
Ec-21_000880     FDIYIDRACGMPYSCTITKVVVRVIGDVGNEGHCSAISEGDSPSNNPVFGLRQEYRTGPE
MSTRG.40198.1    FDFYVDRACGMPYSCTVSKVVVKVFGDVGEQGDCSAISQADSPSNNPVFGLRKEYRTRPD
                 ############################################################


                       1390      1400      1410      1420      1430      1440
                 =========+=========+=========+=========+=========+=========+
Ec-21_000880     SRMDMTSTVMIRVDTLDRFSSEHRAVGYALLAIFMDPSGNQPTNASADGAFLREGNYQVP
MSTRG.40198.1    ERMEMTSTVMVRLDTLDRFSSEHRVVGYALLAIFMDPSGNQPTNASADGAFLREGNYQVP
                 ############################################################


                       1450      1460      1470      1480      1490      1500
                 =========+=========+=========+=========+=========+=========+
Ec-21_000880     IYQKPPPDMRRVTSQSLDRCLRVPCASVLVRVKPASKSSAGKTLSRADVPEDEWKHFGLA
MSTRG.40198.1    LYQKPPPDLRRVTAMSLNNCRRVPCASVLVRVKPASKSSAGKTLGRDDVPEHEWKQFGLA
                 ############################################################


                       1510      1520      1530      1540      1550      1560
                 =========+=========+=========+=========+=========+=========+
Ec-21_000880     PPTPQFSPGVYDNSDLELTDLERQILDLRSKDTYEETVKEAVKLINSPDNPAPGSDSDSD
MSTRG.40198.1    PPIPEFAPGVYDNSDLQLTDLERQILELRSKDKAVEVVGETGKLMKSSDDPAPEYV----
                 #####################################################       


                       1570      1580      1590      1600      1610      1620
                 =========+=========+=========+=========+=========+=========+
Ec-21_000880     DDTLEEWAESMMAKKPTKMVEYHRSEKYRPDQGFSVVVAGLTNMKPPGAFKSARLFKDRP
MSTRG.40198.1    ------------------------------------------------------------
                                                                             


                       1630      1640
                 =========+=========+======
Ec-21_000880     RPGIRQNLDAPRNGNGNDEVVDMGFM
MSTRG.40198.1    ---------------------DEEDN
```

```
Parameters used
Minimum Number Of Sequences For A Conserved Position: 2
Minimum Number Of Sequences For A Flanking Position: 2
Maximum Number Of Contiguous Nonconserved Positions: 8
Minimum Length Of A Block: 10
Allowed Gap Positions: None
Use Similarity Matrices: Yes
```

```
Flank positions of the 6 selected block(s)
Flanks: [919  1166]  [1178  1201]  [1217  1238]  [1240  1260]  [1263  1272]  [1277  1553]  

New number of positions in Ec-21_000880.aa.fasta.trans.aa_ali.fasta-gb:  602  (36% of the original 1646 positions)
```
